# “I belong to nowhere now”: An interpretative phenomenological analysis of Pakistani migrant women’s lived experiences of identity, nostalgia, exclusion and wellbeing in Norway

**DOI:** 10.1177/17455057261484359

**Published:** 2026-09-06

**Authors:** Eisha Mehtab, Gulnaz Anjum, Halina Grzymała-Moszczyńska, Mudassar Aziz

**Affiliations:** 1 Department of Psychology, University of Oslo, Oslo, Norway; 2 Institute of Psychology, Ignatianum University in Krakow, Krakow, Poland; 3 The Norwegian College of Fishery Science, UiT The Arctic University of Norway, Tromsø, Norway

**Keywords:** Pakistani women, migration, identity, nostalgia, postcolonial feminism, Norway, interpretative phenomenological analysis, wellbeing

## Abstract

**Background:**

Migrant women’s health and wellbeing are shaped by intersecting structures of gender, race, religion, and postcoloniality. Pakistani migrant women in Norway occupy a particular intersection of these structures, in which collectivistic kinship expectations, patriarchal norms, and postcolonial inheritance interact with Norwegian institutional gatekeeping. Despite a growing Pakistani-origin population in Norway, little qualitative research has examined how the identity work of recent first-generation Pakistani migrant women relates to their psychological and social wellbeing.

**Objectives:**

This study examined the psychological and social wellbeing implications of identity reconstruction among Pakistani migrant women in Norway, with particular attention to how acculturative challenges, structural exclusion, and gendered nostalgia shape stress, belonging, and adaptation.

**Design:**

Qualitative study using Interpretative Phenomenological Analysis (IPA) with a postcolonial feminist orientation. The reporting of this study conforms to the Consolidated Criteria for Reporting Qualitative Research (COREQ).

**Methods:**

Eleven in-depth semi-structured interviews were conducted with Pakistani migrant women aged 20 or older who had migrated to Norway between 2020 and 2023 and were fluent in Urdu and English. Eight interviews were prioritised for in-depth idiographic IPA case work based on rich, idiographic suitability for analysis, and the remaining three were retained as contextual cases. Informational sufficiency was assessed through ongoing analytic memo-writing. Reflexivity, theory triangulation, and multivocality strengthened methodological rigour.

**Results:**

Five interrelated Group Experiential Themes captured participants’ lived experiences: (1) Resisting and severing gendered expectations, with implications for chronic stress and internalised surveillance; (2) Reconstructing identity in self-exile, with implications for self-efficacy gains and relational isolation; (3) Encountering exclusion in institutions and everyday life, with implications for anxiety, sleep disruption, and stress associated with administrative limbo; (4) Nostalgia, liminality, and limbo, with nostalgia operating as both an emotional regulator and an amplifier of loneliness; and (5) Intergenerational and inter-cohort cultural (dis)identification, with implications for diminished belonging and intra-community alienation.

**Conclusion:**

Identity reconstruction among Pakistani migrant women in Norway is a health-relevant process in which structural exclusion, gendered norms, and cultural nostalgia converge to shape stress, belonging, and agency. The Identity Meaning-Making Model offers a recursive framework with direct implications for culturally responsive mental health care and equity-driven integration policy.

## Plain language summary

Many women move from Pakistan to Norway for study, work, or family. Moving to a new country changes daily life, and it can also change how a woman sees herself. We wanted to understand how recently arrived Pakistani women rebuild their sense of who they are, and how this process affects their mental health and wellbeing. We interviewed eleven Pakistani women who moved to Norway between 2020 and 2023. Each woman took part in one long, private interview and could speak in Urdu or English. We then studied each interview in depth and compared experiences across the group. The women described five connected experiences. First, they let go of old expectations about how they should behave as women, which brought freedom and also guilt and stress. Second, living independently in a new country made many feel more capable and, at the same time, more alone. Third, long waits for residence permits, and schools and employers not recognising their qualifications, caused worry, poor sleep, and a feeling of life being on hold. Fourth, missing home was comforting at times and painful at other times, especially during festivals like Eid. Fifth, some women felt distant from parts of the established Pakistani community in Norway, which deepened their loneliness. Our conclusion is that rebuilding identity after migration is a health matter, not just a cultural one. Health services can help by offering care that understands women’s language, culture, and migration experience. Faster, fairer handling of permits and recognition of qualifications would also protect migrant women’s health. The women’s own strength and creativity were central to how they coped.

## Introduction

Global migration today is both a journey of survival and an expression of dignity and belonging, shaped by widening inequalities, instability, and colonial legacies. In 2020, the International Organization for Migration reported that 281 million people were living as migrants worldwide.^1^ Many people migrate from Pakistan in search of economic opportunity or to escape political and religious insecurity. Gendered and structural inequalities, however, make this movement particularly complex for women. Pakistan consistently ranks among the lowest countries in the world for gender equality. In the 2025 Global Gender Gap Report, Pakistan ranked 148 out of 148 countries.^2^ These rankings reflect low female labour force participation, limited representation in leadership, and persistent restrictions on justice, reproductive autonomy, and mobility.^2^

Layered identities of religion, race, class, and gender compound discrimination when Pakistani women migrate to the Global North, where Islamophobia, racialisation, and linguistic exclusion converge. These intersectional positions have documented consequences for women’s health. Migrant women from racialised backgrounds in Northern Europe report higher psychological distress, depressive symptoms, and lower use of mental health services than majority populations.^3,4^ Structural racism and the chronic stress of recognition denial shape immigrant health beyond what culturalist accounts can explain.^5,6^ For Pakistani migrants in Oslo specifically, comparative data show worse self-rated health and higher psychological distress than ethnic Norwegians.^7^

Colonial legacies deepen these inequalities. British rule institutionalised legal, agrarian, and educational hierarchies whose effects persist in postcolonial Pakistan, contributing to uneven human development within the country, especially along urban-rural, gendered, and regional lines.^8–10^ The colonial experience also produced a collective disillusionment that left many Pakistanis economically constrained at home while culturally idealising the West.^9^ This double bind shapes how women understand both their reasons to leave and their disappointments after arrival.

Pakistani migration to Norway began in the early 1970s with labour migrants who arrived in advance of the 1975 immigration stop.^11^ Unlike some Western European labour migration flows, this movement was not part of a formal bilateral agreement between Norway and Pakistan. It was initiated by individual workers responding to demand for unskilled and semi-skilled labour. Subsequent decades brought family reunification, student migration, and smaller numbers of skilled-worker and asylum-route arrivals, including members of religious minorities such as Ahmadi Muslims.^11^ Pakistani women’s labour market participation has remained disproportionately low. Statistics Norway data indicate a gender employment gap of roughly 40 percentage points among first-generation Pakistani-origin adults, with consequences for household income, social participation, and integration.^11^ Second-generation patterns differ markedly, although discrimination in hiring processes persists even where formal language requirements are met.^12^ For many first-generation Pakistani women, migration to Norway therefore carries expectations of mobility and respect that often give way to renewed marginalisation in work, recognition, and daily life.

### Identity, migration, and recognition

Identity is a dynamic psychological construct that guides behaviour, belonging, memory, and meaning-making.^13,14^ Following an integrative view of identity,^13^ identity can be personal (values, goals, self-esteem), relational (roles and obligations to others), collective (ethnicity, religion, nationality, gender), and material (objects, artefacts, symbols of continuity). These layers are shaped by sociohistorical forces and by the groups with which one is allowed or denied affiliation.^15,16^ We treat these layers as interconnected, drawing on collective and relational identity theory for categorical positions such as gender, religion, and migrant status, and on personal and narrative identity theory for how women narrate continuity and transformation across migration.

Migration disrupts these identity layers by removing the familiar infrastructures through which individuals are recognised and valued. It has been described as a process of estrangement and self-exile,^17^ in which those who cross borders are sorted through hierarchies of race, class, religion, gender, and legality.^18^ For Pakistani women, these hierarchies are not abstract: they determine who is seen as employable, modern, or integrated, and who is made to feel perpetually suspect.

Social identity processes help explain how these hierarchies consolidate. Prejudice and exclusion can be produced and justified through intergroup competition for resources and status,^19^ and migrants’ position in that hierarchy influences their sense of dignity and security.^15,20,21^ Recognition matters because self-worth depends on how one is perceived and allowed to belong.^20^ For migrants this dependence becomes acute, since the institutions and relationships through which recognition is granted have been disrupted. When recognition is withheld, the injury is emotional and embodied, because recognition is a fundamental resource for positive self-regard.^22^ In Norway, Pakistani migrants report persistent barriers in labour market access and credential recognition, experiences of racialised exclusion, and ongoing feelings of powerlessness, all of which concentrate distress and reinforce economic precarity.^23^ Discrimination and gatekeeping in hiring continue to be experienced even when formal language requirements are met, reflecting intersectional stigma tied to race, gender, and religion, including being visibly Muslim and South Asian.^12^

### Nostalgia as affective continuity and health-relevant identity regulator

Nostalgia operates as an affective regulator of identity continuity, with documented links to wellbeing and mental health outcomes. For migrants, nostalgia evokes a deep yearning for past identities, relationships, rhythms, and places. The term joins nostos (homecoming) and algos (pain); early clinical writing framed it as a psychic disturbance associated with grief, melancholia, and loss.^24–26^ More recent work recognises nostalgia as a coping response to displacement that provides emotional continuity during rupture, protects the self against fragmentation, and supports adjustment to dislocation.^26,27^ Nostalgia also allows migrants to defend against frustration and to manage ongoing distress by reconnecting to a coherent sense of self.^28^

For Pakistani women specifically, nostalgia is gendered labour. Women are expected to preserve cultural memory, transmit moral and religious values, and hold together kinship structures even as they navigate isolation, under-recognition, and restricted agency in the host society.^29^ This work of remembering is both protective and costly. It allows continuity, dignity, and rootedness. It also reinforces the sense of loss, longing, and suspended belonging. In this way, nostalgia becomes both an anchor and a wound.

### Liminality, life on hold and women’s belonging

Pakistani migrant women navigate identity within a state of liminality. Ricoeur^30^ distinguishes between idem (sameness, continuity, the expectation to remain recognisably the same) and ipse (the self as transformable, revisable, flexible). Migration forces an ongoing negotiation between these two positions. Women are asked to prove sameness (respectable, family-oriented, non-threatening, willing to adapt) while being pushed to transform (modern, integrated, employable, fluent). The psychic cost of sustaining both demands is high.

Within this negotiation, nostalgia operates as a self-defence mechanism, an attempt to stabilise identity by preserving a coherent narrative across rupture.^26^ Gender, race, religion, and nationhood are not separate axes but co-constituted.^18^ The diasporic guilt of wanting to escape, missing roots, and not belonging anywhere is intensified by the afterlives of colonialism.^18,31^ Pakistani women in Norway inhabit what Bhabha^32^ conceptualises as a Third Space, a zone of in-between existence where they are neither fully anchored in the country of origin nor fully recognised in the host society. In this third space, belonging must be constantly negotiated. Brah^18^ describes the resulting terrain as diaspora space, a politicised arena where inclusion, exclusion, memory, and power are continuously contested. Pakistani women are required to demonstrate cultural legibility to both contexts, authentically Pakistani to family and kin, appropriately modern to Norwegian institutions, while being structurally positioned as suspect in each.

This state is lived as limbo. Migrants can enter an extended period of suspension marked by uncertainty, lack of control, the sense that life has been paused, and that the future is administratively blocked while the present does not feel like home.^33^ For Pakistani women in Norway, this limbo is intensified by specific bureaucratic and acculturative challenges. These include residence permit delays, professional licence recognition through the Directorate of Health (Helsedirektoratet), credential evaluation for foreign degrees through NOKUT, the gendered dimension of family reunification status, and Norwegian language acquisition under unfunded study conditions. While these barriers are not exclusive to Pakistani women, the specific constellation of gendered family expectations, Muslim visibility, and South Asian racialisation gives the experience its particular shape. Tereshchenko^34^ describes this adaptation process as a liminality struggle in which instability, disorientation, and interim existence collide. Women describe being physically present in Norway but temporally caught elsewhere.^33,35^

Liminality therefore has direct implications for women’s health. It is a chronic condition of uncertainty rather than a question of cultural adjustment alone. When recognition is denied in the labour market, when language is used as a gatekeeping tool, and when institutional exclusion fragments self-worth, the result is emotional strain and embodied stress, with effects on anxiety, depressive symptoms, sleep, and somatic complaints documented in migrant cohorts in Northern Europe.^3,7,22,33^ Pakistani migrant women manage this by drawing on continuity (religion, memory, kinship) and by resisting what is imposed on them. This tension between resistance and exhaustion is central to how identity is rebuilt.

### Literature gap and the present study

Existing research on migrant identity, acculturation, and nostalgia tends to treat these themes separately and overlooks intersectional and decolonial perspectives that centre South Asian women’s lived experiences. Within cross-cultural psychology, acculturation frameworks such as Berry’s^36^ have been influential. These frameworks have been critiqued for assuming a linear path between two cultural orientations and for underplaying the emotional, historical, and gendered complexities of migration from collectivistic and postcolonial societies.^5,6^ Within migration studies and postcolonial scholarship, structural and intersectional accounts have been developed but rarely intersect with cross-cultural psychology. Within women’s health research, the embodied consequences of recognition denial and chronic liminality remain under-theorised for South Asian Muslim women in Northern Europe.

This study contributes at the intersection of these three literatures. It asks how Pakistani migrant women in Norway make sense of their identities, nostalgia, and acculturative experiences within intersecting structures of gender, race, and postcoloniality, and how these processes shape their psychological and social wellbeing. Guided by a feminist and decolonial lens, the research asks: how do Pakistani migrant women construct and negotiate identity, belonging, and emotional continuity in a new cultural context? Specifically, it explores how women experience cultural identity and nostalgia post-migration, how they navigate prejudice and marginalisation, and how resistance and memory function as psychosocial strategies for adaptation.

Building on the analysis, this paper develops an Identity Meaning-Making Model that integrates affective nostalgia, postcoloniality, and institutional recognition denial within a single recursive structure. The model is introduced after the empirical findings in the Discussion. Rather than claiming to supersede existing frameworks, the model synthesises affective, postcolonial, and recognition-based perspectives that acculturation and identity integration frameworks have tended to treat in isolation. It offers this synthesis as a heuristic, and it speaks to both social and personal identity, with domains that operate across micro (personal narration), meso (family and community), and macro (institutional recognition) levels.

## Methods

This is a qualitative, exploratory interview study. IPA was the most suitable methodology for this research, since it commits to understanding lived experience and the meaning-making that occurs within it, while taking seriously context, contradiction, and voice. The method embraces complexity, emotional depth, divergence, and convergence, making it possible to explore the layered experiences of migrant South Asian women making space in the Global North. This aligns with the study’s postcolonial feminist lens. IPA also has a rich theoretical basis in phenomenology, hermeneutics, and idiography. We followed the seven-step IPA process developed by Smith, Flowers and Larkin.^37^ The reporting of this study conforms to the Consolidated Criteria for Reporting Qualitative Research (COREQ).^38^ The completed COREQ checklist is provided as Supplementary Material. The study was conducted between June 2024 and July 2025 at the Department of Psychology, University of Oslo, Norway. Data collection took place between December 2024 and May 2025. Data analysis was completed between May and August 2025.

### Philosophical ontology and epistemology

The research is grounded in a constructivist ontology in which realities are socially constructed, fluid, and dynamic rather than fixed or intrinsic. Within the cultural diversity of Pakistan, women take on multiple identities whose meanings are continuously formed and reformed by subjective experiences and societal interactions during migration. The epistemology aligns with interpretivism, with attention to subjective meanings in an intersectional context. Informed by Mohanty’s work on Third World feminism,^39,40^ this study seeks to remain reflexive and contextually attentive, and to avoid epistemic violence by centering migrant women’s own narratives.

### Researchers’ positionality and reflexivity

The study was conducted by EM and supervised by GA. Both are Pakistani women researchers who are themselves immigrants in Norway. This offers an informed insider perspective on the experiences of Pakistani migrant Muslim women navigating life as perceived outsiders. A shared cultural background enabled deep engagement with participants’ narratives and a nuanced understanding of linguistic and social references. This reflexive positionality functioned as a decolonising practice that acknowledges both privilege and limitation in interpreting participants’ voices. Reflexivity was maintained throughout the research process through the IPA framework’s emphasis on self-awareness, theoretical pluralism, and cultural humility, ensuring that personal experience enriched rather than constrained interpretation and that women’s diverse realities remained central. HGM and MA provided additional reflexivity, feedback, and support with the project. Their academic positioning within European institutions as migration researchers allowed for critical distance.

### Research ethics

The study received ethical approval from Sikt, the Norwegian Agency for Shared Services in Education and Research, and the University of Oslo’s Internal Review Board (reference 33196939). Participants were informed about the study aims and procedures, and written consent was obtained prior to participation. Ethical safeguards included a brief post-interview screening (PHQ-4) to monitor potential distress and the provision of follow-up support resources. All data were anonymised, coded, and securely stored on encrypted, password-protected institutional servers with access limited to authorised researchers. The pseudonymisation key was stored separately in TSD (Services for Sensitive Data), in compliance with GDPR and institutional data management standards. Some participants accessed the support resources provided. No identifiable information was collected, and names were anonymised and coded with culturally meaningful flower names in Urdu, with English translations provided in brackets so that readers unfamiliar with Urdu can recognise the meaning. Strong security measures, including encryption, two-factor authentication, and limited access, were enabled throughout.

### Research quality and rigour

The study adheres to Tracy’s eight big-tent criteria for excellent qualitative research,^41^ with attention to relevance, methodological coherence, and ethical integrity. Credibility was supported by thick description, theory triangulation, and multivocality. Transparency was maintained in the Urdu to English translation process to preserve participants’ tone and intent. Resonance emerges from participants’ vicarious and transferable experiences. The study contributes both practically and heuristically, and engages procedural and exiting ethics, including sharing summary findings with participants. Overall, the study demonstrates meaningful coherence by aligning its aims, methods, and interpretations within a decolonial feminist framework.

### Participants and recruitment

The study aimed to recruit Pakistani migrant women meeting three inclusion criteria: being aged 20 or older, having migrated to Norway between 2020 and 2023, and being fluent in Urdu and English. The inclusion criteria were developed during two pilot interviews and refined to support phenomenological homogeneity as IPA requires.^37^ The 2020 to 2023 recruitment window was chosen for three substantive reasons. First, the study sought to capture recent first-generation migration experience in which the rupture with pre-migration life was still actively present in memory and identity work. Second, this window captures arrivals during and after the COVID-19 pandemic, a period in which institutional procedures such as residence permits, credential evaluation, and language schooling were disrupted in distinctive ways. Third, the bounded window supported a phenomenologically meaningful homogeneous sample, with participants sharing salient contextual conditions while differing in personal trajectories. The age criterion of 20 or older was a research ethics decision to ensure adult informed consent and capacity to reflect on migration experience.

Participants were recruited through purposive and snowball sampling, primarily via social media. Most single women had migrated for education or employment, while married women had arrived through family reunification. Two participants identified with Pakistani religious minority backgrounds, and one with a regional ethnic minority background. These positions contributed especially to the analysis of intergenerational and intra-community alienation, since their relationship to Pakistani national identity included pre-migration experiences of religious or ethnic othering.

Eleven interviews were conducted in total. All eleven informed the development of the Group Experiential Themes. Following Smith, Flowers and Larkin,^37^ eight interviews were prioritised for in-depth idiographic IPA case work based on rich, idiographic suitability for analysis. The remaining three interviews were retained as contextual cases that informed the cross-case analysis and the identification of divergent voices. No interview was excluded on the basis of profession or sociodemographic characteristics. Further participant details are provided in Appendix E.

Informational sufficiency was assessed through ongoing analytic memo-writing during interviewing, following Malterud, Siersma and Guassora’s information power framework^42^ and current IPA guidance.^37^ We did not pursue data saturation in the traditional grounded-theory sense, since IPA prioritises depth over breadth and works with small homogeneous samples. By interview eight, new interviews were producing variations rather than additions to the experiential statements clustering toward each emerging Group Experiential Theme. Three further interviews were conducted to confirm informational sufficiency and to incorporate divergent voices, after which recruitment was concluded.

### Procedure

Following ethical approval, two pilot interviews were conducted with single Pakistani women to refine the interview guide (Appendix B). The final semi-structured interviews were organised around eight thematic areas covering background, pre-migration identity, home and nostalgia, migration and adaptation, barriers, identity reconstruction, wellbeing and reflections, and future orientation. Interviews were conducted both online and in person, each lasting approximately 75 minutes. Interviews were one to one, with no third parties present and no repeat interviews, and all women who consented completed their interview. Transcripts were not returned to participants for review. Member reflections were conducted in Spring 2025, in which preliminary themes were shared with consenting participants and their feedback informed the final framing of the findings. Participants received an information sheet and signed consent form (Appendix C) before participation. Interviews were audio-recorded using Diktafon, accompanied by reflexive field notes, and securely stored on Nettskjema. Transcripts, including Urdu to English translations, were downloaded to a password-protected USB and uploaded to TSD. Data were anonymised and pseudonymised using culturally meaningful flower names in Urdu. Part 4 (Migration and Adaptation) elicited several health-relevant accounts, including disrupted sleep during prolonged visa processing, weight changes during early settlement, and persistent anxiety associated with hiring rejections. Part 7 (Wellbeing and Reflections) explicitly probed emotional state, sources of distress, and coping strategies, and produced accounts of loneliness, somatic exhaustion, and grief. After completion of our study, we also maintained follow up contact with some participants who contacted us due to high migration stress.

A brief post-study questionnaire (PHQ-4) was administered solely to monitor participants’ emotional wellbeing as a safeguarding measure. It did not inform thematic analysis. No participant scored in the moderate or severe distress range, although some accepted the follow-up support resources offered.

### Data analysis

**Figure 1.**
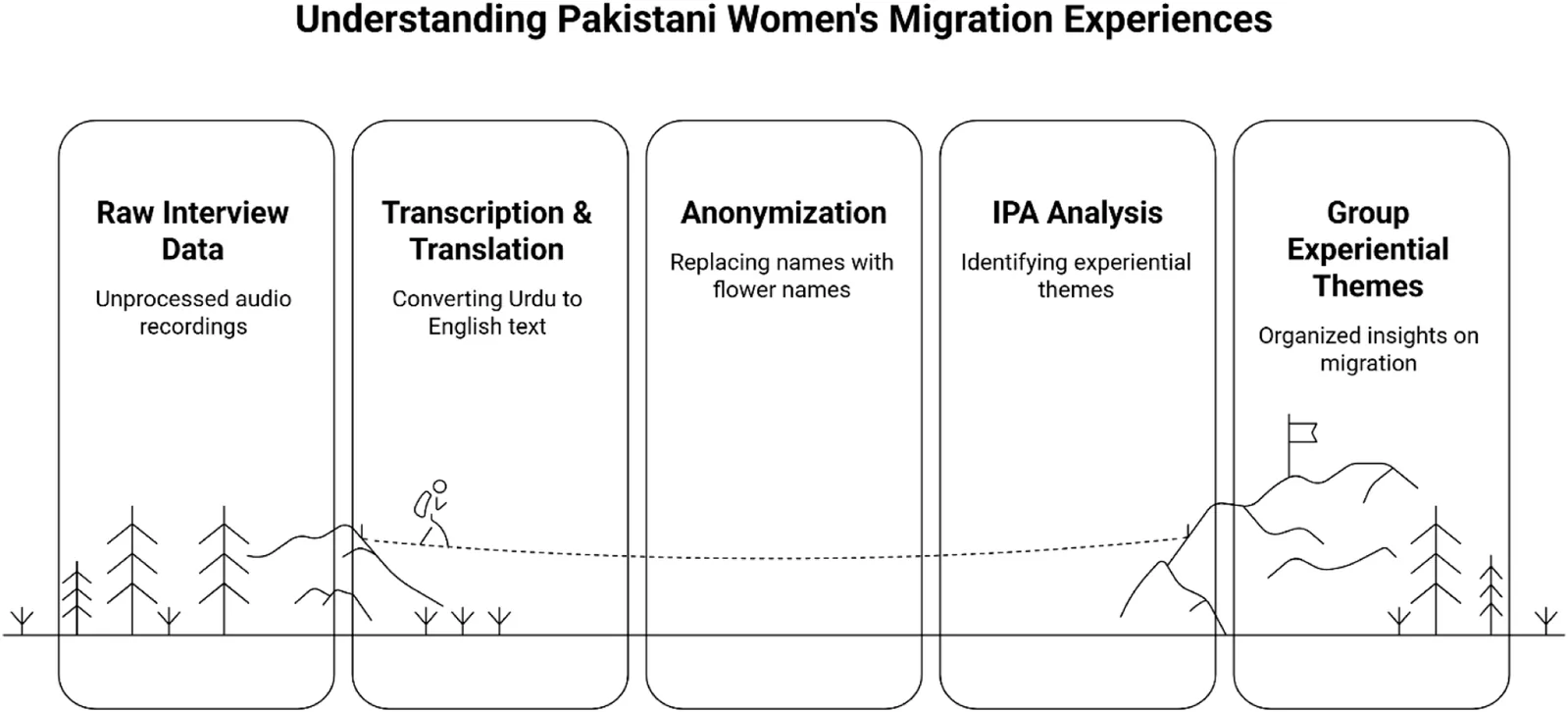
Understanding Pakistani women’s migration experiences. Note. The figure illustrates the stepwise analytic process used to explore Pakistani migrant women’s lived experiences. Beginning with raw audio recordings, interviews were transcribed and translated from Urdu to English, followed by anonymisation through flower-name pseudonyms. Using IPA, experiential themes were identified and refined into five Group Experiential Themes representing organised insights into identity, nostalgia, exclusion, and belonging within the migration journey.

Data analysis followed the seven-step IPA process developed by Smith, Flowers and Larkin^37^ (see Figure 1): familiarisation with each transcript and exploratory note-making (Steps 1 and 2), construction of experiential statements (Step 3), clustering of experiential statements and searching for connections across cases (Steps 4 to 6), culminating in the organisation of Group Experiential Themes (Step 7). Figures illustrating the analytic process are presented in Appendices H to I. EM conducted the primary coding and experiential statement clustering manually in Microsoft Word and Excel, consistent with IPA’s idiographic commitment to close engagement with each case before cross-case analysis. GA and HGM reviewed coding decisions and theme development at the individual case, cross-case, and GET stages, and disagreements were resolved through supervisory discussion. Theoretical concepts were engaged at the interpretive stage and did not pre-determine the themes. Consistent with the qualitative design, no statistical analyses were performed. The PHQ-4 described under Procedure served safeguarding purposes only and was not analysed.

## Results

Five Group Experiential Themes (GETs) emerged from the analysis: (a) Resisting and severing gendered expectations; (b) Reconstructing identity in self-exile; (c) Encountering exclusion in institutions and everyday life; (d) Nostalgia, liminality, and limbo; and (e) Intergenerational and inter-cohort cultural (dis)identification. Detailed tabular summaries of all five GETs were created. Divergent participant voices are highlighted at the end of each table to mark them clearly, following indications of high-quality IPA reporting.^37^ Each GET below opens with a short framing line that names the theoretical constructs that inform interpretation, and closes with a brief statement of the psychosocial wellbeing implications of the experiences described.

### GET 1: Resisting and severing gendered expectations

This GET is read through Mohanty’s account of women from the Global South as active agents of resistance,^39,40^ Ricoeur’s distinction between idem (sameness, the expectation of remaining recognisably oneself) and ipse (the transformable self),^30^ and Hall’s notion of identity as production rather than essence.^43^ Pakistani women in this study describe gender policing and control, met with strict boundaries, limitations, and expectations in Pakistan. Post-migration, they actively recognise, resist, and sever gendered boundaries to establish a refined identity within a new culture. Most participants describe a sense of restriction, internalised thinking systems, and patriarchal influence.

**Chambeli:** I think Pakistani women, they are very much bound in the self and society-made boundaries. We have come out of society, but we are still in the self-made boundaries. There is a difference between can or want. I can do everything that I want. But do I want? Right? So, it’s not you who’s going to decide for me. There’s no wrong in having your traditional values. But then those traditional values should stick with you because you want them, not that you’re expected to do them.

Chambeli differentiates between can and want and points to how women in Pakistan can find it difficult to recognise their want (desire) because of gendered control, despite the presence of their can (capability). This internalised surveillance suggests that external control becomes a means of regulating authentic preferences. It points to how gender norms operate beyond physical space. Sumbul and Champa describe hyper-surveillance into personal affairs. This communal surveillance disrupts their sense of belonging within the home and within their bodies.

Yasmin offers a divergent voice that complicates a simple narrative of severance. She describes missing certain situational advantages that Pakistani patriarchal culture grants to women in public space, including the Aurat card, a colloquial reference to deferential treatment.

**Yasmin:** I think I was more empowered in Pakistan because I was more in control of my finances, more in control of my decisions. Because in Pakistan, you know, we have that ‘ladies, ladies’ idea, the ‘Aurat card’ (Woman card). I really love that. We have this thing in Pakistan. Here it’s nothing like that. So, I really miss that.

Yasmin’s account illustrates the ambivalence of severance. The same gendered system that constrains can also confer pockets of advantage that are then mourned. Women reconstruct their gender identity after severance through resistance that takes many forms, including questioning inherited norms, redefining the womanhood they were taught, and slowly dismantling the weight of communal surveillance.

The wellbeing signature of this theme is chronic vigilance: pre-migration internalised surveillance and post-migration severance work together as ongoing stressors. Participants describe ongoing self-monitoring, intrusive worry about communal judgement, and a relational labour of severance that is cognitively and emotionally demanding. These patterns correspond to the documented links between gendered surveillance, anxiety, and somatic complaints among South Asian women.^3,6^

### GET 2: Reconstructing identity in self-exile

This GET is read through Bhabha’s notion of the Third Space,^32^ Hall’s account of identity as positional,^43^ and Ricoeur’s ipse as the transformable self.^30^ Women describe identifying new ways of experiencing selfhood by renegotiating autonomy, reinventing roles, building hybrid identities through cultural reorientation, and challenging cultural and gendered internalisations. Their social identity, national identity, vocational identity, and cultural or religious identity were prominent. Many women described being pampered and reserved in Pakistan, pointing to a delayed individuation. This also marks their self-exile, in which they estrange themselves from home to settle abroad, producing a voluntary displacement.

**Nargis:** In Pakistan, I was so pampered. Yeah. Dependent on my family for everything. My brother, he has to do everything for me. Because I have done things which I never thought in my life I would do that. I think I feel myself more strong here.

Champa describes the importance of being able to ask for help, which challenges the self-silencing pattern of patriarchal socialisation she had previously been culturally conditioned to. Her capacity to show vulnerability becomes a source of strength.

**Champa:** And this was because of my society that I was a very reserved person and I thought that I have to sort everything myself, I will not get any help. And if I remember the person I was three to four years before, I was very, like, I don’t need help, I just accept everything that I see. I don’t need to ask questions. This is a very important thing. I don’t need to ask questions. But in Norway, I started asking questions, asking for help.

Motiya’s belongingness and self-worth were tied to being mobile, financially independent, and autonomous. Her account also points to how much dependence there was on women’s male counterparts, such as fathers and brothers, to do basic things for women. This delayed self-sufficiency and signalled a withdrawal of trust in women and the culture she belongs to.

**Motiya:** But when I moved here, I felt empowered in a way that you know in Pakistan I always had to wait for my father or someone to come and take me to places. Here, I travel alone whether it’s day and it’s night, it doesn’t matter. And when I started working here, it was paying really well. So I was able to buy stuff and do things for the family. It was a very, very huge thing for me. A very huge accomplishment, if you might say.

These women navigate a complex and dynamic process of identity reconstruction. Women shed inherited constraints and stage revivals of self on their own terms, showing both identity continuation and transformation. Several participants also described a renewed spiritual identity in Norway, in which religious practice ceased to be performative and became self-chosen; we develop this point in the Discussion.

For wellbeing, the autonomy gained through self-exile cuts both ways. Participants describe enhanced self-efficacy, mobility, and decision-making capacity, which support psychological wellbeing. They also describe loneliness, the loss of relational support, and the cognitive load of learning self-sufficiency without familiar scaffolds. This dual pattern aligns with research showing that empowerment among migrant women is relational rather than purely individual.^4,44^

### GET 3: Encountering exclusion in institutions and everyday life

This GET is read through Honneth’s recognition theory^22^ and Brah’s diaspora space,^18^ including racialised gatekeeping that converts language and credentials into instruments of power.

#### Subtheme 1: Discrimination hidden behind language rigidity

Most women described being held back by Norwegian language requirements. Women feel alienated and isolated by a double-bind in which they must choose between meeting immediate survival demands such as paid work, study, or care work, and investing time in Norwegian language acquisition without any guarantee of qualified employment afterwards. Future instability prevents present investment. Laleh describes her dilemma:

**Laleh:** And during my master’s, I was busy with that and job applications. I was thinking if I get the job, I will start learning. I did not want to learn Norwegian if I don’t know if I’m going to live in Norway or not. That would be a waste of time.

Motiya describes a situation in which she was working at her odd job and spoke in English to a Norwegian customer. The account points to resistance to multiculturalism and an insistence on linguistic assimilation as moral citizenship, particularly from older Norwegians. A similar experience was shared by Nargis.

**Motiya:** She was old and she said that ‘When you are living in Norway and you expect to deal with us and all, you should learn the language. Even if we can speak English, we are not going to speak English with you, we expect you to speak Norwegian with us.’

Migrants are not asked to speak Norwegian. They are required to speak fluently, as Nargis remarks, and discrimination is hidden in the form of expecting a specific local accent even in English, as experienced by Sumbul. These expectations create a power imbalance that protects the status quo and produces exclusion.

**Nargis:** Why the Norwegian should be necessary or compulsory here? I mean I can adjust myself with English as well. That’s a factor where I think they force you to learn a new language and not just learn, speak good, speak fluently!

Norwegian language ability becomes a measure of worth and inclusion. In Honneth’s terms,^22^ this is misrecognition operating through language. Linguistic rigidity is not itself gendered, but it is converted by the host society into a class and racial issue. Only sufficient money can buy expensive language courses, and even fluency as a South Asian woman is met with indirect but powerful discrimination, in line with Norwegian evidence on accent and racial gatekeeping.^12,45^

#### Subtheme 2: Systematic institutions of exclusion

Many women’s experiences of migration were overshadowed by exclusion and discrimination, most of them related to career, work, and employment. Helplessness and a state of limbo were lived through when navigating institutional challenges and performative inclusion.

Nargis is exhausted by hiring managers always having an excuse for her rejection. Her student-focused life led her to find value in contributing through work after education. Repeated invalidation of her effort affects her self-worth.

**Nargis:** It’s very challenging because sometimes they already have decided that internal candidate. Experience is almost like mandatory here. How can you get experience being a fresh graduate? That is always a problem.

Gulab points to how skilled migrant women have to work jobs below their qualifications (see Appendix E for participants’ professions, education, and current occupations). Given the limited odd-job culture in Pakistan, especially for women, this downgrade is experienced as a status shock that disrupts dignity even when accommodated later.

Visas and permits operated as a brutal systemic barrier. Champa was unable to visit her dying mother due to an eight-month student visa delay. She arrived in Pakistan only for the funeral. Her powerlessness leads her to question the inhumane system, and the grief in exile is amplified by institutional indifference despite procedural compliance.

**Champa:** I was granted my visa just one week before, and I was waiting like seven to eight months. Sometimes I think about it that if I had my visa before I could have met my mother too. Sometimes, I really want to ask them what should I do, what is my fault? So this is, I think this is very inhuman behaviour. I can say it.

Visa delays make women constantly think of their temporality, in which migrant lives are made to wait, to be stalled, and to be devalued.

The health consequences of this theme are the most concrete in the study. Recognition denial operates as a chronic stressor with documented links to anxiety, depressive symptoms, and sleep disruption among migrants in Northern Europe.^3,7,22,33^ Participants describe disrupted sleep during prolonged visa processing, recurring rumination over hiring rejections, and grief compounded by administrative delay. The credential and licensing process produces what we describe in the Discussion as temporal injustice, with embodied consequences for daily functioning. GET 4 turns to how this externally imposed waiting is lived from the inside.

### GET 4: Nostalgia, liminality and limbo

This GET is read through Ritivoi’s account of nostalgia as continuity work,^26^ Akhtar^46^ and Lijtmaer^47^ on migrant nostalgia and mourning, Tereshchenko on the liminality struggle,^34^ and Anjum and colleagues on life and mental health in limbo.^33,35^

Because GET 3 and GET 4 sit close together, we mark their boundary explicitly. GET 3 concerns administrative limbo: the externally imposed suspension produced by visas, permits, and credential systems. GET 4 concerns two related but distinct experiences through which that suspension, and migration more broadly, is lived from within. Existential liminality names the felt condition of being in between, no longer anchored in Pakistan and not fully at home in Norway. Nostalgic longing names the affective orientation toward a remembered past that accompanies this in-betweenness. Administrative limbo is a structural condition, liminality is its lived temporality, and nostalgia is the emotional register in which the past is revisited, revised, and mourned. The accounts below show how the three interlock without collapsing into one another.

Most women evaluated nostalgia as a way of locating belonging in an alienated space and culture. Memory and nostalgia were sources of dislocation and disruption during migration, often reopening wounds as women faced liminality with little progress. Their nostalgia was sometimes experienced as stable emotional grounding and more often as a paradox of homesickness and fragmentation. Yasmin remains grounded and reimagines her past life through creative expression, which helps her preserve identity and archive sensory remembrance.

**Yasmin:** You can guess from this that I somehow still feel connected with the streets, with the vibe of my childhood. So there is a painting in front of me that I painted. It’s a building from Old City. Sometimes I follow recipes that we have eaten from the small stalls of Old City. My nostalgia is not that of mountains or sceneries. My nostalgia is traffic, big cities, big buildings.

Yasmin’s creative archive is continuity work. Her paintings, recipes, and traffic memories stabilise a sense of self across rupture. Nilofer, in contrast, experiences nostalgia as existential homelessness, which could destabilise identity over time. When festivities like Eid come, nostalgia becomes overwhelming, and avoidance and rational distancing help.

**Nilofer:** I feel like now I have no home. So, I miss my family a lot sometimes, especially on Eid. I keep myself busy so that I will not miss them anymore.

Nostalgia also operated as a gendered paradox. Escaping patriarchal control also meant losing care and attention, with collectivist warmth giving way to isolating individualism.

**Laleh:** The freedom itself is also a very big cage for us. We are never actually given that. And in Pakistan, you are the centre of attention in so many ways. Now nobody is actually watching you. That was also a problem. And the loneliness itself was a very challenging problem.

Laleh’s description of freedom as a very big cage captures the paradox of self-exile. Mostly, nostalgia was experienced as not wanting to feel nostalgic for a home that reminded participants of why they had left. Pakistani migrant women evaluate their nostalgia in diverse ways. It is not a stark barrier to integration, but it does signal a displacement, reminding women of the reasons for their escape. Their nostalgic yearning is folded into forces of liminality and limbo.

Nostalgia’s significance for wellbeing is accordingly double-edged. Sensory and creative memory practices support emotional regulation and continuity. Loss-focused nostalgia, especially around festivals and absent kin, amplifies loneliness and grief. These patterns echo work that associates nostalgia deficits with diminished social wellbeing^48^ while also showing nostalgia’s protective role.^27^

### GET 5: Intergenerational and inter-cohort cultural (dis)identification

This GET is read through Hall’s notion of diasporic différance,^43^ Anzaldúa’s borderlands,^49^ and the broader intersectional literature on diaspora and difference.^18,50^ Participants make sense of their lived experiences of migration by rejecting or disidentifying with the culture they come from. This rejection matters because it reflects what to take forward and what aspects of culture are worth identifying with, both for themselves and intergenerationally. Women expressed internal conflicts, contradictions, and dual consciousness, so they could proceed with a dignified cultural continuity. What is described here as intergenerational difference is also an inter-cohort difference shaped by the different conditions under which earlier and recent Pakistani migrants entered Norway.^11,12^

Champa describes the experience first-hand. Her professional licence (autorisasjon) to practise as a health worker, issued by the Directorate of Health (Helsedirektoratet), was delayed for more than two years. She fears that this delay reflects Norwegian immigration authorities’ perception of all Pakistanis as fraudulent or suspect. Being humiliated and belittled by host country authorities, on top of being punished for others’ wrongdoings, generates strong cultural dissonance.

**Champa:** But the reason that I’m not getting my licence is that there are so many people who have done fraud to the health directorate and for their accommodation, for their experience and everything. And that’s why they are taking too much time. So this one reason has affected me too much that I don’t feel proud of my culture and my country. If I talk about UDI and also health directorate, they discriminate too, now. It’s very belittling, you feel very humiliated sometimes.

Yasmin notes a diaspora and intra-community disconnect. This produces a lack of emotional connection with their own people, with consequences for the belongingness of newly arrived migrants. When the familiarity of culture feels close, the imposed disconnection of one’s own cultural group can isolate new migrants further.

**Yasmin:** What I found is like there’s a huge difference, not just the generation difference, but there’s a huge cultural difference from first generation to the Pakistanis who came now in the last 20 or 10 years. Second generation of Pakistanis in Norway are living in the same mindset that Pakistan is very poor.

These women experienced significant disappointments because of betrayal and a lack of recognition of their agency from their culture. Despite being complex and painful, this is a layered detachment. Even the place to which they have escaped may not be the redeeming alternative they had imagined.

What this layered detachment costs women is protective connection. Cultural dissonance and intra-community alienation diminish belonging and weaken the buffering effects of co-ethnic social support that the migrant mental health literature typically describes.^3,4,7^ Participants describe shame, the affective burden of representing a stigmatised national group, and a sense of double exclusion from both the host society and parts of the established diaspora.

## Discussion

This section interprets the GETs through the Identity Meaning-Making Model and the wider literature. We first introduce the model as the empirical contribution of the study, then we synthesise the findings under four cross-cutting themes, and we then turn to health implications, strengths, limitations, future research, and practical implications.

### The identity meaning-making model

**Figure 2.**
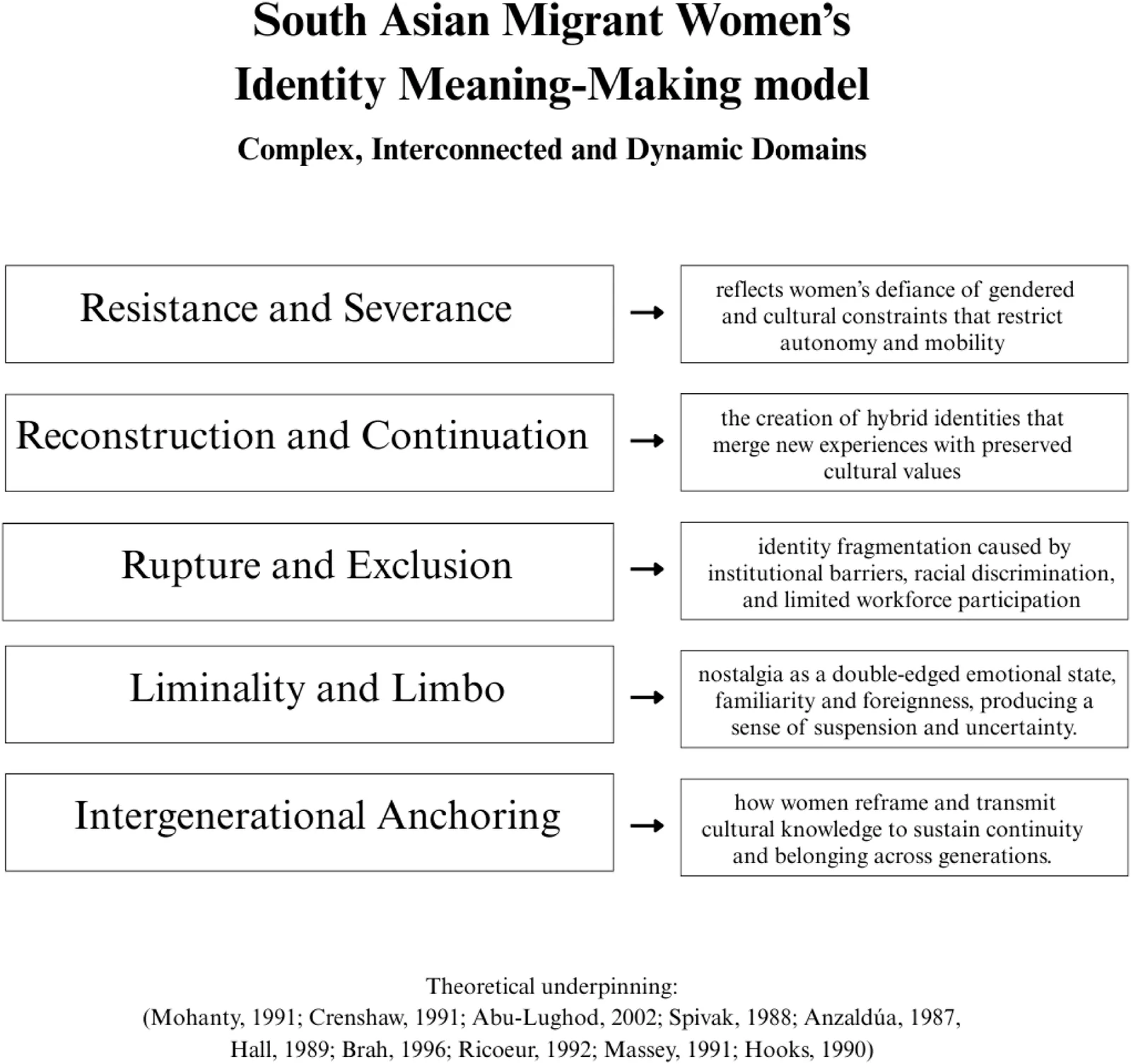
Proposed theoretical model of identity meaning-making of South Asian migrant women. Note. Figure 2 The figure depicts migrant women's five dynamic and interconnected domains of identity reconstruction derived from the analysis.

The Identity Meaning-Making Model (Figure 2) comprises five recursive and interconnected domains derived from the GETs. The domains are processes rather than outcomes. Women circulate among them over time, and the model is intended as a heuristic for capturing simultaneity rather than as a stage model.

The five domains are: Resistance and Severance, in which women defy gendered and cultural expectations and enact psychic breaks from what is framed as home; Reconstruction and Continuation, in which women assemble hybrid identities by preserving selected values and practices while reworking self-definition; Rupture and Exclusion, in which identity is fragmented by institutional barriers such as linguistic gatekeeping, racial discrimination, and blocked access to employment; Liminality and Limbo, in which women inhabit the affective condition of being suspended between past and present, belonging and unbelonging, safety and uncertainty, where nostalgia becomes both protective and painful; and Intergenerational Anchoring, in which women renegotiate culture through care, deciding what to retain, what to discard, and what to pass on to their children.

The domains operate at multiple levels. Resistance and Severance is primarily personal and relational. Reconstruction and Continuation is personal, vocational, and religious. Rupture and Exclusion is structural and intergroup; Liminality and Limbo is affective and existential; Intergenerational Anchoring is relational and collective.

Theoretical underpinnings are integrated across domains. Ricoeur’s idem and ipse^30^ informs Resistance and Severance and Reconstruction and Continuation. Hall and Bhabha^32,43^ inform Reconstruction and Continuation through the positional and Third Space framings. Honneth and Brah^18,22^ inform Rupture and Exclusion through structural recognition denial. Ritivoi, Akhtar, Lijtmaer, and Tereshchenko^26,34,46,47^ inform Liminality and Limbo through affective nostalgia and the liminality struggle. Anzaldúa, Anthias, and Crenshaw^49–51^ inform Intergenerational Anchoring through intersectional and borderlands framings. The model’s contribution is synthetic rather than singular. It brings together affective, postcolonial, and recognition-based perspectives that acculturation and identity integration frameworks have tended to treat in isolation, and it offers this synthesis as a heuristic and empirically grounded map of how the five processes interlock, rather than as a replacement for existing frameworks.

### Identity as negotiated continuity and rupture

For Pakistani migrant women in Norway, identity emerges as a negotiation between continuity and rupture. GET 1 demonstrates how women re-evaluate selfhood by resisting internalised patriarchal norms and communal surveillance. This resonates with Mohanty’s argument that women from the Global South are active agents of resistance, rather than passive subjects of cultural constraint.^39,40^ Participants’ reflections on the difference between can and want reveal internalised self-regulation that mirrors the disciplinary logic of patriarchal control. Their acts of resistance, including questioning inherited norms, redefining respectable femininity, and choosing autonomy, constitute what Hall^43^ calls identity as a production, always in process and contingent on power relations.

The study extends these ideas through Ricoeur’s distinction between idem and ipse.^30^ Women’s transitions reflect both the persistence of self-continuity and the transformation of agency through new contexts. Their post-migration identities blend resistance and reclamation, consciously severing restrictive expectations while redefining femininity in intercultural space; the ambivalence some women voiced about lost situational advantages shows that severance is mourned as well as chosen. This embodies a liminality between pre-migration subordination and post-migration autonomy.^19^

### Reconstructing autonomy and self-exile

GET 2 highlights how autonomy, once limited by familial dependence, becomes a central marker of self-realisation abroad. Migration produces delayed individuation. Women accustomed to protection and deference in Pakistan now learn self-sufficiency, decision-making, and financial responsibility. For many, this self-exile is both emancipatory and isolating. Empowerment is relational, emerging from re-learning interdependence in unfamiliar settings, rather than only from freedom from control.^4^ Several women experienced a renewed spiritual identity in Norway. Religion ceased to be performative and instead became what Mahmood^52^ terms embodied agency. Autonomy was also gendered through motherhood and vocational roles. Migrant mothers often face a moral hierarchy in which care work overshadows professional identity,^53^ illustrating how acculturation is mediated by gendered expectations.^54^ Structural barriers, including the devaluation of foreign qualifications^55^ and limited mobility, compounded these struggles and produced lower life satisfaction^56^ and a sense of liminality between opportunity and constraint.

### Nostalgia and liminality as affective spaces

GET 4 demonstrates that nostalgia operates as both a coping resource and a source of dissonance. Women’s sensory memories of food, sounds, and familiar streets anchor identity across temporal and spatial divides, an instance of what Brah^18^ calls lived locality. This aligns with research on nostalgia’s role in continuity and emotional regulation.^27,57^ Under conditions of bureaucratic delay and exclusion, nostalgia also amplifies loss, producing spatial dislocation.^58^

Participants’ ambivalence, including missing Pakistan’s communal warmth while cherishing autonomy in Norway, illustrates nostalgia as a double consciousness. This validates Ritivoi’s argument that adaptation depends on perceiving identity as continuous despite rupture.^26^ In this study, nostalgia sometimes hindered adjustment by reactivating loss rather than offering cohesion. This echoes the association between nostalgia deficits and diminished social wellbeing.^48^ For some, nostalgia became a reminder of why they left, confirming Ali’s observation that memory can reproduce awareness of past hierarchies rather than comfort.^29^ Nostalgia reveals migration as a state of self-imposed exile, a space of both emancipation and mourning.

### Structural exclusion and recognition

GET 3 highlights systemic exclusion embedded in everyday and institutional practices. Linguistic rigidity exemplifies symbolic domination. Language proficiency becomes a moral measure of citizenship. Similar findings in Norwegian contexts show how linguistic and cultural assimilation remain implicit requirements for belonging.^12,45,59^ Women’s accounts of accent discrimination and credential discounting illustrate how power operates through classification.^15,21,60^ These experiences expose the persistence of social dominance hierarchies^61^ in which native Norwegians retain structural privilege.

The Identity Meaning-Making Model conceptualises these findings as cycles of recognition and misrecognition. Delays in work permits, non-transferable qualifications, and precarious residence statuses produce temporal injustice. Women are trapped in administrative waiting, a suspended life that devalues their time and contribution. These experiences confirm Honneth’s claim that recognition is essential for self-realisation.^22^ When recognition is withheld, women internalise institutional disregard as diminished self-worth. These results support broader evidence that Norwegian bureaucratic structures, although ostensibly egalitarian, perpetuate racialised and gendered exclusion with embodied health consequences.^7,23,62^

### Cultural (dis)identification and diaspora politics

GET 5 portrays cultural disidentification as an adaptive and painful process. Women selectively retained religious and familial values while rejecting patriarchal authority and systemic inefficiency. This corresponds with Hall’s notion of identity as positional rather than essential.^43^ Cultural pride was undermined by perceived stigma associated with Pakistani nationality, particularly when bureaucratic suspicion of fraud delayed licences or visas. Such collective penalties reinforce internalised shame and what Jost and Major describe as system justification, in which migrants rationalise inequalities to preserve coherence.^63^

Diaspora fragmentation also shaped identity politics. Many women felt alienated from earlier Pakistani cohorts in Norway, who appeared assimilated and dismissive of recent arrivals. This echoes Hall’s concept of diasporic différance^43^ in which difference within communities destabilises belonging. Others critiqued Western individualism and neoliberal efficiency, revealing ambivalence toward both origin and destination. Migration produced hybrid identities marked by pragmatic cultural continuity and emotional ambivalence, a negotiation of dignity across borders.^64^

### Health implications and culturally responsive care

Taken together, the findings show that identity reconstruction has direct implications for the health and wellbeing of Pakistani migrant women in Norway. Chronic stress under gendered surveillance, isolation in autonomy gain, anxiety and sleep disruption under administrative limbo, and the dual valence of nostalgia together describe a psychosocial environment with known consequences for depressive symptoms, anxiety, and somatic complaints among migrant women.^3,4,6,7^ These findings underline the need for culturally responsive mental health care that engages diasporic affect rather than pathologising it.^65^ Services should recognise the protective dimensions of nostalgia and cultural continuity, and they should address the structural sources of distress rather than locating distress only in individual coping deficits. For Pakistani migrant women specifically, gender-aware and language-inclusive support is critical, since both gender norms and linguistic gatekeeping are central to the experiences described in this study.

### Strengths and significance

A significant strength of this study is its focus on intersectionality and on the often subaltern voices of South Asian Muslim migrant women of colour. The study challenges Eurocentric assumptions of acculturation, argues against assimilationist framings, and contributes to decolonising perspectives on migration psychology. This orientation gave rise to the Identity Meaning-Making Model. The study examines how Pakistani and Norwegian value systems intersect and where they require deliberate alignment. It informs equity-driven integration and inclusion policy for Norway, and offers reflections for Pakistani women on managing alienation and anger through sustained meaning-making. Nostalgia studies remain limited in the context of migration, especially for reducing prejudice and increasing emotional stability. The study contributes to this literature.

Reflexivity functioned as a methodological strength throughout the analysis and supported critical awareness of how the researchers’ positionalities shaped interpretation. Deep engagement with participants’ contrasting experiences of agency, belonging, and privilege allowed underlying structural inequities to emerge. Emotional attunement during data interpretation enhanced sensitivity to narratives of loss, exclusion, and resilience. This reflexive engagement was strengthened by methodological safeguards including theory triangulation and multivocality, and revealed how institutional rather than only interpersonal discrimination structures migrant women’s everyday realities in Norway.

## Limitations

Several limitations are noted. Our findings on autonomy and professional exclusion reflect the experiences of a highly educated and recent migrant sample, primarily women holding master’s degrees. Women who arrive through family reunification without independent professional credentials, women in lower-skilled or informal labour pathways, and women in non-urban settlement contexts may negotiate identity differently. These groups are priorities for future research. Participants were not compensated for their time due to a lack of funds. The findings may not be generalisable to all Pakistani migrant women in Norway. Subjectivity of experience is central to IPA, and interpretations reflect the capacity of participants to articulate their experiences^66^ and of the researcher to bracket preconceptions.^67^ Future studies could conduct fewer interviews with a shorter interview guide for deeper idiographic work.

A further limitation concerns our own position. The shared insider positionality of EM and GA, as Pakistani migrant women in Norway, clearly facilitated recruitment, trust, linguistic nuance, and depth of disclosure. The same closeness may also have shaped the analysis. Participants may have emphasised accounts they expected co-national women to understand, and we may have heard accounts of gendered constraint with particular readiness. We addressed this through reflexive journaling, deliberate attention to divergent voices, and critical checking by HGM and MA, whose different positionalities supported alternative readings. Interpretive shaping cannot be fully excluded, and we report it transparently as an inherent condition of insider qualitative research rather than treating it as eliminable.

## Future research

Future research should extend the Identity Meaning-Making Model through participatory and mixed-method approaches that integrate visual, narrative, and quantitative analyses to capture the layered nature of migrant identity work. Participatory action research can co-create knowledge with migrant women and extend the relevance of the work beyond academia. Comparative studies should include second-generation Pakistanis, Norwegian-Pakistanis, queer South Asian migrants, and interfaith or mixed-heritage families to explore intersecting positionalities and transnational belonging. Cross-cultural replication across South Asian contexts such as India, Bangladesh, Nepal, and Sri Lanka would test the model’s conceptual robustness and identify culturally specific versus shared features of migrant identity negotiation.

Further studies should examine the perspectives of majority-group Norwegians to understand how perceptions of migrants shape integration and institutional prejudice. Intervention-based research could evaluate targeted programmes, including free in-person Norwegian language instruction through community-led initiatives, workshops on labour rights and anti-discrimination for migrants, and educational modules for employers addressing bias in hiring and credential recognition. Experimental or longitudinal studies could test the potential of nostalgia-based interventions to reduce intergroup prejudice and enhance empathy between migrants and host communities.^68,69^

Representation of samples from the Global South remains uneven across the women’s health literature. Bibliometric and systematic mapping of women’s reproductive, mental, and physical health show that empirical scholarship continues to draw disproportionately on populations in high-income Northern contexts, while women in South Asia and the broader Global South appear far less often as primary subjects of research.^70,71^ This concentration shapes which experiences become legible as women’s health concerns and which remain peripheral. Migrant women in Europe sit at the intersection of two patterns of underrepresentation. They originate from contexts that are themselves understudied, and they live in transitions that cross physical, mental, and reproductive domains that tend to be researched in isolation rather than as an interconnected lived experience. Justice-oriented analyses of whose voices structure migration scholarship document a parallel fragmentation. Global South perspectives appear less frequently, and integrated accounts that link bodily, emotional, and reproductive transitions to the social conditions of migration remain rare.^72^ More integrated research that treats physical, mental, and reproductive health of migrant women in Europe as connected rather than parallel domains would give better representation to needs that have remained at the margins of European health scholarship. The present study contributes to addressing this gap by centering Pakistani migrant women’s own narratives, treating identity work, nostalgia, institutional exclusion, and wellbeing as inseparable from the broader gendered and migratory conditions in which they unfold.

## Practical implications and policy recommendations

The findings underscore the need for structural rather than individualised approaches to integration.

### Institutional reform

Norwegian institutions should shift from assimilationist frameworks toward intercultural inclusion, with gender-sensitive and equity-driven policies. Credential recognition processes through NOKUT, UDI, and the Directorate of Health should be reviewed for speed, transparency, and equity, particularly for foreign-trained health workers. Visa and permit processing systems require acceleration and transparency to reduce temporal precarity and emotional distress. Free Norwegian language courses should be expanded for migrant women that are currently not eligible for free courses, like those who are not co-habiting with or married to EU/EEA partners, especially during their job-seeking period. Employer-sponsored language training should be offered to qualified candidates. Policy reform could also reconsider unpaid internships and develop merit-based opportunities aligned with emerging fields of study.

### Community-level support

At the community level, incentivised social programmes that foster cross-cultural engagement and dialogue between Norwegian and migrant populations could mitigate isolation and enhance mutual understanding. For Pakistani women specifically, participation in language cafés, civic education, and peer support groups can strengthen agency and belonging. Initiatives grounded in decolonial and gender-aware perspectives can promote equitable integration and contribute to migrant women’s psychosocial wellbeing.

## Conclusion

This study set out to understand how recently migrated Pakistani women in Norway reconstruct identity, and what that reconstruction means for their health. The answer, across five Group Experiential Themes, is that identity work is health work. Gendered severance, autonomy gain, institutional exclusion, liminal suspension, and cultural (dis)identification converge on the same psychosocial terrain: chronic stress, disrupted sleep, loneliness, and grief, alongside real gains in self-efficacy, agency, and self-chosen belonging. The Identity Meaning-Making Model organises these processes into a heuristic map that clinicians, service planners, and policymakers can use. Two implications follow. Mental health services should offer culturally responsive, gender-aware, and language-inclusive care that engages diasporic affect, including nostalgia, as a resource rather than a symptom. Integration policy should treat credential recognition, permit processing, and language access as health interventions, because the temporal injustice they currently produce is lived by migrant women as embodied distress.

## Supplemental material

Supplemental Material - “I belong to nowhere now”: An interpretative phenomenological analysis of Pakistani migrant Women’s lived experiences of identity, nostalgia, exclusion and wellbeing in Norway Supplemental Material for “I belong to nowhere now”: An interpretative phenomenological analysis of Pakistani migrant Women’s lived experiences of identity, nostalgia, exclusion and wellbeing in Norway by Eisha Mehtab, Gulnaz Anjum, Halina Grzymała-Moszczyńska, Mudassar Aziz in Women's Health.

## Data Availability

Supplementary information and additional details have been provided with the submission. Additional anonymised data will be provided on reasonable request. Please contact the corresponding author. *
