# Supplementary material for: “I belong to nowhere now”: An interpretative phenomenological analysis of Pakistani migrant women’s lived experiences of identity, nostalgia, exclusion and wellbeing in Norway
Source: Womens Health (Lond). 2026 Sep 6;22:17455057261484359. doi: 10.1177/17455057261484359 (PMC13554598; doi:10.1177/17455057261484359)
Supplement: Supplementary file 1 [file sj-pdf-1-whe-10.1177_17455057261484359.pdf]

Supplementary Material

Appendix A – Ethical evaluation of research project from IRB and Sikt

UiO : University of Oslo
Faculty of Social Sciences – Departement of Psychology

Eisha Mehtab
Gulnaz Anjum

Ref.number: 33196939
Date: 4 December 2024

Ethical evaluation of research project

Your project, "A Qualitative Phenomenological Account of Pakistani Diaspora Women's Identity and Resilience Formation: Rediscovering Nostalgia and Diaspora Culture" has been ethically evaluated by the Department of Psychology's internal research ethics committee.

After the evaluation The Department of Psychology's internal research ethics committee recommend the project.

Sincerely yours, on behalf of the Committee,

Professor Silje Endresen Reme, Head of Committee
Members of the Department of Psychology's Research Ethics Committee
https://www.uio.no/for-ansatte/enhetssider/sv/psi/psi-eng/internal-ethics-committee/index.html

Postal address:
E-mail:
www.uio.no

Assessment of processing of personal data

Print 11.02.2025

Reference number	Assessment type	Date
954099	Standard	11.02.2025

Title
A Qualitative Phenomenological Account of Pakistani Diaspora Women: Identity and Resilience Formation in Diaspora Culture

Institution responsible for the project
University of Oslo / Faculty of Social Sciences / Department of Psychology

Project leader
Gulnaz Anjum

Student
Eisha Mehtab

Processing time
11.11.2024 – 31.07.2025

Categories of personal data
General
Special

Legal basis
Consent, cf. GDPR Article 6(1)(a)
Explicit consent, cf. GDPR Article 9(2)(a)

Notification Form

> The processing of personal data is lawful, as long as it is carried out as stated in the notification form. The legal basis is valid until 31.07.2025.

ABOUT OUR ASSESSMENT
Data Protection Services has an agreement with the institution where you are a student or researcher. As part of this agreement, we provide guidance so that the processing of personal data in your project is lawful and complies with data protection legislation. We have now assessed that you have a legal basis to process the personal data.

TYPE OF DATA AND LEGAL BASIS
The project will process special categories of personal data about ethnicity.

The legal basis for processing personal data will be consent from the data subject, as stated in Article 6(1)(a) of the General Data Protection Regulation (GDPR). The data subject will give explicit consent to the processing of special categories of personal data. The conditions in Article 9(2)(a) of the GDPR will therefore be met, and the prohibition on processing special categories will not apply.

FOLLOW YOUR INSTITUTION'S GUIDELINES
You must store, send and secure the collected data in accordance with your institution's guidelines. This means that you must use data processors (and the like) that your institution has an agreement with (ie cloud storage provider, online survey tool, or video conferencing platform).

Our assessment presupposes that the project will meet the requirements of accuracy (Article 5(1)(d)), integrity and confidentiality (Article 5(1)(f)) and security (Article 32) when processing personal data.

NOTIFY CHANGES
If you intend to make changes to the processing of personal data in this project, it may be necessary to notify us. This is done by updating the information registered in the Notification Form and sending it in. On our website we explain which changes must be notified. Wait until you receive a reply from us before you carry out the changes: https://sikt.no/en/notify-changes-notification-form

FOLLOW-UP OF THE PROJECT
We will follow up at the planned end date to determine whether the processing of personal data has been concluded. In long duration projects, we will follow up every other year.

Good luck with the project!

Appendix B – Interview guide

Process and Structure of the Interview: The interview will follow a semi-structured format, combining open-ended questions with tailored prompts to guide the conversation. We will begin with a disclaimer, and ice-breakers (casual questions about the weather and personal hobbies) to establish a relaxed and non-intrusive atmosphere. The core of the interview will explore themes related to participants' migration experiences, including their reasons for moving, life before and after the move, definitions of identity, relationships, and coping mechanisms. The structure allows flexibility, enabling participants to share their thoughts freely while we guide the discussion through prompts when necessary. A debriefing and additional space for further thoughts will conclude the interview, ensuring participants feel heard and supported throughout the process.

Interview Disclaimer: "Thank you for agreeing to participate in this interview. As a reminder, your participation is completely voluntary, and you may choose to skip any questions or stop the interview at any time. This interview is in your control. Take however long you need to answer a question. If something is not popping up suddenly, please, by all means, take your time. Let me know if you don't know what a question means, or a certain word means. All the information you provide will be kept confidential and anonymised to ensure your privacy. Please don't disclose very sensitive personal information that could compromise your anonymity. These questions ask about your background in Pakistan, your childhood, and your move to Norway, life before the move and after, your reasons for moving, the moving process and how it made you feel, your definitions of identity, your relationships of support, activities before and after the move, your situation now and how you take care of yourself. As a fellow Diaspora Pakistani woman, I understand that some topics may feel personal or sensitive, and you are encouraged to share only what you feel comfortable with. Please feel free to stop me if you need clarifications on a question in Urdu, and if you feel like you want to talk in Urdu to explain something, please do."

Ice-breakers: Ask about something they enjoy doing in their free time. (This prompt will allow participants to share something positive and non-intrusive, creating a relaxed atmosphere without diving into personal or sensitive topics. It helps establish a sense of ease before moving into more detailed or challenging parts of the interview.)

Part 1: Background and Arrival to Norway
Purpose: Establish rapport and gather personal, migration-related background.

Q. Can you tell me about yourself— your age, where you're from, what your family background is, when you moved, and what led you to move to Norway?

Q. Describe your life here; what keeps you occupied, how do you spend your time? Where do you work?

Q. What were your first impressions of Norway when you arrived? Can you describe any moments that felt particularly striking or memorable?

Part 2: Pre-migration identity, cultural roots
Purpose: Explore identity before migration and the role of cultural upbringing

Q. What was your life like before you moved to Norway?

Q. Before you migrated, how did you see yourself as a woman in your hometown?
Prompt: What expectations—cultural, familial, or societal—shaped your sense of identity?

Q. Did you ever feel empowered, or were there moments you felt restricted by any norms?

Part 3: Home, Childhood, Nostalgia and Identity Conceptualisations
Purpose: Experience past as they remember it

Q. What is home for you?

Q. If someone asked you to describe your identity, where would you begin?
If asked for explanation: Identity be linked to religion, country, beliefs, and value systems

Q. When I say words like 'nostalgia' (purani yad) 'childhood' (bachpan) and 'home' (ghar) what memories or feelings come to mind for you?' What do you feel most nostalgic about when it comes to your childhood?
Q. Do you miss it or wish to go back or not return?

Part 4: Adaptation, Settling In, Prejudice, Possible Barriers and Exclusion
Purpose: Understand experiences in Norway as a Pakistani female immigrant.

Q. What has been the hardest part of adjusting to life in Norway? Can you describe a moment or experience that felt particularly challenging?

Q. How has learning Norwegian been for you?

Q. Have you ever felt treated unfairly (work, community) in Norway because of your background or identity? What happened, and how did it affect you?

Q. Do you feel Norway's society allows space for your cultural identity to thrive, or does it pressure you to conform? Why?

Q. What changes do you think could help reduce prejudice against Pakistani women in Norway?
Probe: Can you share any examples from your own life or from what you've observed in the community? What do you wish you had support in?

Q. Do you feel like you belong in Norway now? Why or why not?

Part 5: Identity Negotiation and Preservation
Purpose: Examine how participants navigate identity in a new cultural context.

Q. How do you balance your Pakistani identity with living in Norway?

Q. What aspects of being a woman feel most significant to you? How do you feel about feminism?

Q. Do you feel your identity as a Pakistani woman has changed since moving to Norway? If so, how?
Probe: Do you feel more empowered, constrained, or something else altogether?
→ personality or identity?

Q. What habits, hobbies and activities did you engage yourself with before? Do you continue them now?

Part 6: Experiences with migration, identity and decolonisation
Purpose: See how does it all ties together

Q. How has moving to Norway changed the way you see Pakistan and Pakistani culture?

Q. Have you ever felt pressure to align with Western norms, like how you speak, dress, or behave? How have you managed these pressures?

Q. Do you feel a sense of pride in your cultural roots, or have there been moments of doubt or conflict?

Q. How has the presence or absence of familial support impacted your ability to integrate into Norwegian society while maintaining connections to your Pakistani cultural roots?

Part 7: Self-care, Social Networks and Support

Purpose: Understand the role of community in identity preservation and resilience.

Q. Can you tell me about your network of friends or community in Norway? How do these relationships affect your sense of belonging, how do they help you?
→ do you still feel you need more support?

Q. What do you do to take care of yourself, emotionally and mentally?
Prompt: Any artistic or cultural practices and what inspires them? (performing arts (dance, music and theatre), poetry, painting, applying mehndi, pottery, embroidery, knitting, stitching, cooking, calligraphy, socio-political activism)

Part 8: Reflection and Closing

Purpose: Provide space for personal reflection and additional insights.

Q. What do you think people in Pakistan or Norway misunderstand most about your experience?
Q. If you could change one thing about your experience of living in Norway, what would it be?
Q. Is there anything else you would like to share that we haven't discussed?

After the interview: A free schedule will be maintained after the interviews in case the participants have more to share or elaborate on.

Debriefing:
"Thank you so much for your time today. Your insights are incredibly valuable and will help us better understand the experiences of Pakistani women in the diaspora. The purpose of this interview was to explore migration, identity, and integration, and your responses will make a significant contribution to this research.

I want to reassure you that your privacy and confidentiality are very important to us. Your responses will be pseudonymized, meaning that your name will be replaced with a code, and the list linking participants to codes will be kept separate. Later, all identifying details will be removed, making it impossible to re-identify you. The data will first be pseudonymized and later anonymized, following standard procedures.

If at any point you would like to withdraw your data, we can remove your responses. Please feel free to reach out if you have any further thoughts or questions about the research. If any sensitive topics were touched on during the interview, I can provide information about the support services that are available to you.

Just a reminder, you are free to withdraw your participation at any time before the data is anonymized. After anonymization, which will happen approximately four weeks from now, your data will be kept in aggregate form for research purposes and it won't be possible to link it back

to you. If you would like to amend or remove any of your responses before anonymization, you are welcome to contact me.

Once again, thank you for your participation. You’ve been heard, and we really appreciate your contribution to this study."

Appendix C – Information guide and consent form

Are you interested in taking part in the research project
"A qualitative phenomenological account of Pakistani diaspora women's identity and resilience formation: Rediscovering Nostalgia, Patience and Pakistani Culture"?

Purpose of the project
You are invited to participate in a research project whose main purpose is to understand how diaspora Pakistani women make their place, space, identity and presence pre and post-migration in Norway. This is an exploration and understanding of diaspora Pakistani women's experiences in preserving their identity and hence aims to help migrants by talking about the importance of identity for brown migrant women. The project aims to promote well-being by understanding the capacity of women to take care of themselves and gives insight into how cultures and practises from the Global South make space in the Global North.

The research involves conducting interviews and is carried out as part of a master's thesis. Your personal data will not be used for any other purposes.

Which institution is responsible for the research project?
The University of Oslo is solely responsible for the project (data controller).

Why are you being asked to participate?
You are being asked to participate if you are a Pakistani woman who has migrated to Norway to give insight into your identity, culture, practises, your past (childhood) and present to understand the migration and integration process to Norway and how your identity plays a role in your daily life. There will be a total of 10 women who will be interviewed.

What does participation involve for you?
This is a semi-structured interview with the purpose of understanding your migration reasons, processes, feelings, and your past and childhood life in Pakistan. The questions will be about your move to Norway, life before the move and after, reasons for moving, the moving process and how it made you feel, your definitions of identity, your relationships of support, activities before and after the move, your situation now and how do you take care of yourself in times of stress. These will help us understand how you used to identify yourself previously and how has that changed (or remained unchanged) after you moved to Norway. While this study involves minimal risk and has no known risks, certain aspects such as emotional discomfort or privacy concerns could arise. Privacy risks have been mitigated by following the data privacy regulations of GDPR, stating in clear language how participants have the right to withdraw their consent and have their data deleted at any time before anonymization. Before the interview, they will be asked not to share information that is specific or private. Some questions may trigger affective

reactions, which you have **complete freedom not to answer.** The primary investigator (Eisha Mehtab) and the supervisor (Dr Gulnaz Anjum) have trained in Mental Health and Human Rights Info's (MHHRI) Gender-Based Violence Manual for Women (https://www.hhri.org/) with certification, as well as in Teaching Recovery Techniques from the Children and War Foundation and is equipped to handle such reactions. Moreover, participants will also be given leaflets post-interview from MHHRI's resources, more specifically, the Women's Toolbox (https://www.hhri.org/women-manual/), which focuses on grounding techniques, stabilisation exercises, the window of tolerance and other tools in case the participants need them.

You are also reminded before the interview that you have the complete right to withdraw from the interview at any time without giving a reason, and the right to withdraw before anonymisation, and not to share any specific, personally sensitive or identifiable information.

The interview will take approximately 1 hour (60 minutes). You will be requested to fill in a consent form. Your answers will be recorded electronically and will only be used for transcription purposes so that the researcher does not forget to note down key information, which will later be used to analyse in qualitative research software. Once all data is transcribed and anonymized, the digital recording of your interview will be deleted.

The participants will be compensated with a coffee or food for their time and valuable contribution to this project. Since this is snowball sampling, it will involve you sharing the researcher's contact information with others who might be interested. Potential participants can then choose to contact the researcher directly if they wish to participate.

Participation is voluntary

Participation in the project is voluntary. You may withdraw your consent and have your data deleted at any time before anonymization. After anonymization, data will be kept in aggregate form for research purposes, making it impossible to link back to you. Anonymization is expected to occur approximately four weeks later. There are no known negative consequences for you if you choose not to participate or later decide to withdraw. If you are part of any groups or working anywhere, participation in this research will not affect anything since we are only interested in your lived experience and opinions from a personal perspective.

Your personal privacy – how we will store and use your personal data

We will only use your personal data for the purpose(s) specified here and we will process your personal data in accordance with data protection legislation (the GDPR). You can exercise your GDPR rights by providing your unique code, which will be retained solely by the researchers and

the participants. This code will be the only means of identifying you if you wish to exercise your rights under GDPR, such as accessing or deleting your data.

- Only the researcher and supervisors will have access to your personal data. Pseudonymization will involve replacing names with codes and retaining a separate list of participant identities. Anonymization removes all identifying details, making re-identification impossible. In this study, data will first be pseudonymized and later anonymized, following standard procedures. The pseudonymization key linking participant identities to coded data will be stored in TSD, separate from the main dataset. Strong security measures include encryption, two-factor authentication, and limited access restricted to authorized personnel. If someone would like to withdraw their data, we can do "quote matching" to drop the quotes from those participants.
- The data will be stored in an encrypted and password-protected folder and server and no unauthorised person can gain access to your personal data. Your names will be replaced with a unique code and any other data (like recordings) will be stored in a different folder so that it becomes impossible to match.

The data would be managed and analysed through the qualitative software NVivo. UiO will allow recording via Diktofon, and process and store data on TSD on its cloud service. All data files will be securely stored in UiO’s TSD (Services for Sensitive Data), ensuring compliance with institutional guidelines for data security. The data analysis will be done in the researcher’s NVivo account.
You will not be recognisable in publications. If any information will be published, it might be quotes or phrases that you have said, or reworded, and they will have no association with who said it.

What will happen to your personal data at the end of the research project?
The planned end date of the project is July 2025. Your personal data and any digital transcripts will be archived at the end of the research project. Once anonymized, the data will be analyzed to identify themes and patterns. Results will be reported in aggregate, ensuring that individual responses remain unidentifiable. The data will be stored for 5 years and then securely deleted. Nothing will be archived later.

Your rights
So long as you can be identified in the collected data, you have the right to:
- access the personal data that is being processed about you
- request that your personal data be deleted
- request that incorrect personal data about you be corrected/rectified
- receive a copy of your personal data (data portability), and

- send a complaint to the Norwegian Data Protection Authority regarding the processing of your personal data

What gives us the right to process your personal data?
We will process your personal data based on your consent.
Based on an agreement with the University of Oslo, The Data Protection Services of Sikt – Norwegian Agency for Shared Services in Education and Research has assessed that the processing of personal data in this project meets requirements in data protection legislation.

Where can I find out more?
If you have questions about the project or want to exercise your rights, contact:
- the Supervisor, Gulnaz Anjum at gulnaz.anjum@psykologi.uio.no
- the Student Researcher, Eisha Mehtab at eisham@uio.no
- Our Data Protection Officer at the University of Oslo: Roger Markgraf-Bye at personvernombud@uio.no

If you have questions about how data protection has been assessed in this project by Sikt, contact:
- email: (personverntjenester@sikt.no) or by telephone: +47 73 98 40 40.

Yours sincerely,

____

Eisha Mehtab
(Researcher/Student)

____

Gulnaz Anjum
(Supervisor)

Consent form
I have received and understood information about the project **"A visual-qualitative phenomenological account of Pakistani diaspora women's identity and resilience formation: Rediscovering Nostalgia, Patience and Pakistani Culture"** and have been given the opportunity to ask questions.

I give consent:
- ☐ to participate in the interview
- ☐ use the content and information I give for analysis
- ☐ for the researcher to give information about me in this project

- ☐ for my personal data to be stored till the end of the project (tentatively July 2025)
- ☐ for my anonymized data to be used for my thesis and research paper

____

(Signed by participant and date)

Appendix D – Data management plan

Sikt

DATA MANAGEMENT PLAN

A Qualitative Phenomenological Account of Pakistani Diaspora Women: Identity and Resilience Formation in Diaspora Culture

This research project is part of a master's thesis in the psychology master's program of Global-MINDS at Psykologisk institutt (PSI) at the University of Oslo. This research will be conducted by the student Eisha Mehtab, supervised by Associate Professor Gulnaz Anjum. This research looks into phenomenological accounts of diaspora Pakistani women with a focus on their experiences of the past, like nostalgia, childhood, and how they use cultural or artistic practises in the present that aid in their identity (re)formation (e.g., identity continuation, attaining new forms of identity, and letting go of older identity). The thesis examines indigenous Global South theories on post-colonialist, decolonising, and feminist perspectives on the lived experiences of Pakistani diaspora women living in Norway.

Disciplines
Social Science

Research responsible institution
Universitetet i Oslo / Det samfunnsvitenskapelige fakultet / Psykologisk institutt

Project duration
11.11.2024 — 31.07.2025

Scientific purpose
How do Pakistani diaspora migrant women reconstruct their identities, preserve their cultures and navigate prejudice in Norway, while balancing nostalgia and the challenges of acculturation? Through this, we aim to answer some secondary research questions broken down from the overarching main research question. Q1. What are diaspora pakistani women's experiences of identity preservation, continuation and negotiation pre- and post-migration? Q2. How does nostalgia (purani yaad) and childhood (bachpan) influence their sense of identity and belonging in a new cultural context? How do they evaluate nostalgia? Q3. How do they navigate prejudice in their everyday life? The core of the interview will explore themes related to participants' migration experiences, including their reasons for moving, life before and after the move, definitions of identity, relationships, and coping mechanisms.

Utility
The research aids social psychological research relating to migration, diaspora, identity and cultural topics. This aids specifically researchers in South Asia or the Global South in learning about migration in the Global North or Scandinavia. This also aids Norwegian and Pakistani psychology organisations aiding in migration and integration aid, for example, IOM Norway. The thesis has these objectives: - Explore and understand diaspora Pakistani women's experiences of identity preservation, continuation and (re)formation (both before and after migration). - Prevent marginalisation by redefining a strong self-identity for Pakistani migrants - Promote well-being by understanding the capacity of migrants to self-protect - Insight into how cultures and practises from the global south interlink into the global north due to migration - Insight into feminist and decolonial perspectives about migration, identity and the past and the present - Deliver a narrative of the struggle, resilience, and progress of Pakistani women

Does the project involve research on humans?
Yes

Describe the considerations or measures taken to safeguard the integrity and privacy of the participants
Ethical considerations: This project will remain strictly social psychology-focused and include no diagnostic or clinical aspects. The study will not involve risks; some questions may trigger affective reactions, which the participant has complete freedom not to answer. However, for questions that may trigger affective reactions, the primary researcher/investigator (Eisha Mehtab) and the supervisor (Dr. Gulnaz Anjum) have trained in Mental Health and Human Rights Info's (MHHRI) Gender-Based Violence Manual for Women (https://www.hhri.org/) with certification, as well as the researcher has trained in Teaching Recovery Techniques from the Children and War Foundation and is equipped to handle such reactions. She is also currently being supervised by Dr Nora Sveaass (Clinical Psychologist and Professor Emeritus at PSI, UiO) at MHHRI as an intern, who will guide how to ensure participant protection. There are, however, no known harms to the research, but if it comes to emotional outbursts, or if the participant feels unease or discomfort, the researcher is trained to work in these situations and has the required certifications. Moreover, participants will also be given leaflets post-interview from MHHRI's resources, more specifically, the Women's Toolbox (https://www.hhri.org/women-manual/), which focuses on grounding techniques, stabilisation exercises, the window of tolerance and other tools in case the participants need them. As with any studies working with interviews, some people may perceive some of the questions as confusing because of their open-ended nature. We will make sure to remove these confusions by reiterating the questions and objectives. Moreover, we will run a mock interview to better prepare for any additional preparedness. Questions in the interview deal with participants' identity as women, their experiences of nostalgia, homeland, childhood, and notions of patience, and other resilience

Yes

Categories of personal data
General

Does the data collection include data on vulnerable groups?
Yes

Number of individuals in the dataset
11

Does the data collection include confidential or graded information?
Yes

Are there any other reasons why the data collection needs extra protection?
No

Security class
Confidential

Comment
The data collection and data material is classified as red, the data will be carefully guarded with strict access regulations and will be at all times transferred and stored at a secure server (TSD) as per the University of Oslo's regulations. The data will first go through Nettskjema-Diktafon for the recordings and through the auto-transcription with AutoText. All data files will be securely stored in UiO's TSD (Services for Sensitive Data), ensuring compliance with institutional guidelines for data security. The data will be transcribed automatically through AI (AutoText /Whisper from UiO) or in Nettskjema through autotranscription and then be put back in TSD and kept there as long as it contains personal information. Direct personal information will be removed during transcription. Next, the data will be analyzed manually/through NVivo on the TSD platform. The data will stay permanently on TSD and not be exported. Moreover, no personal details of the participants are being recorded other than their voice recording, which will be completely removed after data analysis in NVivo. TSD supports the installation of certain software, including NVivo for qualitative data analysis. This requires that the software be approved for use within the secure environment which will be the case for our analysis. The data will be stored in encrypted, password-protected folders on a secure server of TSD. Only authorized personnel (the student and the supervisor) will have access to the data. The pseudonymization key linking participant identities to coded data will be stored in TSD, separate from the main dataset. Strong security measures include encryption, two-factor authentication, and limited access restricted to authorized personnel. While unique codes along with pseudonymization reduce the risk of re-identification, we will have implemented strong security measures to minimise any chance of unauthorised access or matching of personal information. Additionally, access to unique codes will be restricted and carefully monitored, following GDPR and data protection regulations.

Data collection period
11.11.2024 — 31.01.2025

Collection devices
- Sound recorder/dictaphone, institution
- UiO Nettskjema

Data quality
The semi-structured interviews will follow an interview guide, with prompts and guidance questions. This is developed and attached with this application and it includes pre and post interview disclaimers and remarks.

Method
Interview, Recording

Description
These will be one-to-one interviews, taken place in spaces where the women feel most comfortable and safe. Participants have the option to deny a recorded interview and still participate in the study by allowing the researcher to take handwritten notes. Otherwise, they will consent for recording the interviews.

Size
100000 MB

Format
doc, m4a

Software
TSD, Nvivo, Nettskjema, Dictaphone app, AutoText (Whisper OpenAI) (all relating to UiO)

Comment

The data will first go through Nettskjema-Diktafon for the recordings and through the auto-transcription with AutoText. All data files will be securely stored in UiO's TSD (Services for Sensitive Data), ensuring compliance with institutional guidelines for data security. The data will be transcribed automatically through AI (AutoText /Whisper from UiO) or in Nettskjema through autotranscription and then be put back in TSD and kept there as long as it contains personal information. Direct personal information will be removed during transcription. Next, the data will be analyzed manually/through NVivo on the TSD platform. The data will stay permanently on TSD and not be exported. Moreover, no personal details of the participants are being recorded other than their voice recording, which will be completely removed after data analysis in NVivo. TSD supports the installation of certain software, including NVivo for qualitative data analysis. This requires that the software be approved for use within the secure environment which will be the case for our analysis. The data will be stored in encrypted, password-protected folders on a secure server of TSD. Only authorized personnel (the student and the supervisor) will have access to the data. The pseudonymization key linking participant identities to coded data will be stored in TSD, separate from the main dataset. Strong security measures include encryption, two-factor authentication, and limited access restricted to authorized personnel. While unique codes along with pseudonymization reduce the risk of re-identification, we will have implemented strong security measures to minimise any chance of unauthorised access or matching of personal information. Additionally, access to unique codes will be restricted and carefully monitored, following GDPR and data protection regulations.

Naming conventions

Folders in TSD: There will be 1 folder, where the 4 sub-folders will have: 1) Audio 2) contact information, personal information, anonymisation key 3) transcriptions and 4) completed anonymised transcriptions

Storage

- TSD (Services for Sensitive Data)

Transfer

- Unit FileSender

Comment

The data will first go through Nettskjema-Diktafon for the recordings and through the auto-transcription with AutoText. All data files will be securely stored in UiO's TSD (Services for Sensitive Data), ensuring compliance with institutional guidelines for data security. The data will be transcribed automatically through AI (AutoText /Whisper from UiO) or in Nettskjema through autotranscription and then be put back in TSD and kept there as long as it contains personal information. Direct personal information will be removed during transcription. Next, the data will be analyzed manually/through NVivo on the TSD platform. The data will stay permanently on TSD and not be exported. Moreover, no personal details of the participants are being recorded other than their voice recording, which will be completely removed after data analysis in NVivo. TSD supports the installation of certain software, including NVivo for qualitative data analysis. This requires that the software be approved for use within the secure environment which will be the case for our analysis. The data will be stored in encrypted, password-protected folders on a secure server of TSD. Only authorized personnel (the student and the supervisor) will have access to the data. The pseudonymization key linking participant identities to coded data will be stored in TSD, separate from the main dataset. Strong security measures include encryption, two-factor authentication, and limited access restricted to authorized personnel. While unique codes along with pseudonymization reduce the risk of re-identification, we will have implemented strong security measures to minimise any chance of unauthorised access or matching of personal information. Additionally, access to unique codes will be restricted and carefully monitored, following GDPR and data protection regulations.

Archiving

No

Appendix E – Research Participants Table (*n*=11)

Table 1

Participant Information and Demographics

Pseudonym Name (and English translation)	***Age***	***Profession / Highest Education***	***Single/ Married***	***Interview Mode***	***Arrived in Norway***	***Current occupation***
Woman 1 Chambeli (Jasmine)	27	Nutrition / Master	Single	In-person	2021	Healthcare provider
Woman 2 Motiya (Arabian Jasmine)	30	Accounting / Master	Married, 1 child	Online	2021	Homemaker / Unemployed
Woman 3 Nargis (Daffodils)	26	Medical Physics / Master	Single	In-person	2022	Fast food restaurant
Woman 4 Champa (Frangipani)	28	Molecular Medicine / Master	Married	Online	2022	Grocery shop
Woman 5 Laleh (Tulip)	27	Environmental Engineering / Master	Single	Online	2022	Associate Technical Professional
Woman 6 Sunbal (Salmalia)	25	Biotechnology / Master	Single	Online	2022	Food industry
Woman 7 Yasmin (Poet's jasmine)	40	Journalism / Master	Married	Online	2021	Journalist
Woman 8 Nilofer (Lotus)	27	Veterinarian/Animal Science / Master	Single	In-person	2022	Server

Woman 9 Gulab (Rose)	32	Information Technology	Single	In-person	2019/ 2021	Grocery Assistant
Woman 10 Sumbul (Hyacinth)	27	Information Technology	Married	Online	2023	Norwegian Oil Company
Woman 11 Zinia (Daisy)	33	Information Technology/Logistics	Married, 1 child	Online	2019	Logistics

Note: The pseudonyms are flower names in Urdu, chosen for cultural resonance with the participants. The English translations are provided in brackets so that readers unfamiliar with Urdu can recognise the meaning. The translation has no analytic role.
